# Editorial: Plant Molecular Farming: Fast, Scalable, Cheap, Sustainable

**DOI:** 10.3389/fpls.2016.01148

**Published:** 2016-08-03

**Authors:** Domenico De Martinis, Edward P. Rybicki, Kazuhito Fujiyama, Rosella Franconi, Eugenio Benvenuto

**Affiliations:** ^1^ENEA Italian National Agency for New Technologies, Energy and Sustainable Economic DevelopmentRome, Italy; ^2^Biopharming Research Unit, Department of Molecular and Cell Biology, University of Cape TownCape Town, South Africa; ^3^Osaka UniversityOsaka, Japan

**Keywords:** plant molecular farming, biopharmaceuticals, metabolic engineering, recombinant protein, biobetter, genetic engineering, transient expression, plant factory

The transfer of genes into plants, that was achieved in the early 80's, paved the way for the exploitation of the potential of plant genetic engineering, to add novel agronomic traits and/or to design plants as factories for high added value molecules. For this latter area of research, the term “Molecular Farming” was coined because major crops like maize and tobacco were originally used basically for pharma applications.

In this research topic we have tried to gather together the scientific community working on the concept of plant biofactories: this has eventually resulted in a comprehensive display of studies (33 papers from the Americas, Europe, South Africa, India, Australia, Japan, and China) that approach the complexity of producing desired molecules in plants and plant cells, covering the topic from small, but tricky, metabolites to large chimeric proteins.

To develop plant-based “green biofactory” implies advantages over the more conventional cell factories based on animal cells or microbial cultures, when considering the investment and managing costs of fermenters. Nevertheless, when dealing with any biofactory, some challenges remain the same: the feature of the product to be obtained, the engineering of the host, and the production and purification steps that may cause more than “just a headache.”

The studies describe several different approaches to understanding how to boost production of the desired product by molecular engineering (Diamos et al.; Xu et al.; Gurkok et al.; Mercx et al., Dhar et al.) or via biochemical or environmental stimuli (Fujiuchi et al.; Huang et al.; Jiang et al.); how to better store or deliver the desired product (Ceresoli et al.; Passeri et al.; Weichert et al.; Alfano et al.); how to make the product more stable (Mandal et al.; Dicker et al.; Kunert and Pillay); and how to obtain a better purification yield (Sainsbury et al.; Buyel et al.) and better performance (Hofbauer et al.; Matoba) of the molecule.

Thus, although yield, stability, and quality of the molecules may vary among different systems, plants are strongly competitive on a case-to-case basis, and both the molecular design and the plasticity in place and time of production may provide distinct advantages (e.g., use of cell suspensions: Corbin et al.; Santos et al., roots: Häkkinen et al.; Chen et al. or by transient expression rather than stable transformation: Alkanaimsh et al.; Westerhof et al.). For these reasons *engineering the plant factory for the production of biologics and small-molecule medicines* attracts scientists and technologists for the intriguing features of low production cost, product safety, easy scale-up and the possibility to produce “biobetters.”

Molecules that are currently being produced in plants exploit only a little of the immense potential to produce natural compounds (Pulice et al.; Andre et al.), as well as nutritional supplements such as vitamins, carbohydrates and biopolymers (see also previous references) and industrial and pharmaceutical proteins.

The latest products described here promise to provide tools to tackle serious medical challenges, from chronic ones (e.g. Celiac disease, Viegas et al., and Prostate Cancer, Sarkar et al.) to dangerous infections with pandemic potential, such as SARS (Demurtas et al.), influenza (Mbewana et al.), malaria (Spiegel et al.) as well as Salmonella (Miletic et al.). Interestingly, this last panel of publications highlights the modularity of molecular engineering systems that could be platforms for genetic engineering and provision of fast and scalable systems to be used in response to new outbreaks of highly infectious diseases.

Convergence among disciplines as distant as plant physiology and pharmacology and, more recently, the “-omics” sciences, as well as bioinformatics and nanotechnology, increases the options for research on the plant cell factory. Once suitably engineered, a plant is possibly the cheapest and easiest eukaryotic system to be adapted to production of pharmaceuticals, as they can be bred with simple know-how, and grown using only simple nutrients, water and light.

These approaches suggest a future, modular approach to protein design that could represent a new trend in the field (De Paoli et al., 2016) “Farming for Pharming” of biologics and small-molecule medicines is a challenging area of plant biotechnology that may break the limits of current standard production technologies. Market approval of “Elelyso” in 2012 (Protalix/Pfizer, recombinant Glucocerebrosidase produced in carrot cells for treatment of a rare disease) and the recent apparent success in fighting Ebola virus with plant-made antibodies put a spotlight on the enormous potential of next generation plant-made medicines, made especially in the name of the guiding principle of reduction of costs: these will help reduce disparities in health rights as well as tools to guarantee adequate health protection in developing countries (Hinman and McKinlay, 2015; Folayan et al., 2016).

## Author contributions

All authors contributed equally to the manuscript, within their role as editors of the topic.

### Conflict of interest statement

The authors declare that the research was conducted in the absence of any commercial or financial relationships that could be construed as a potential conflict of interest.
